# Supplementary material for: The mediating role of stakeholders on green banking practices and bank’s performance: The case of a developing nation
Source: PLoS One. 2024 May 21;19(5):e0300585. doi: 10.1371/journal.pone.0300585 (PMC11108172; doi:10.1371/journal.pone.0300585)
Supplement: Attachment — Submitted filename: Response to Reviewers.docx [file pone.0300585.s002.docx]

Dear Editor,

PLOS One.

**Subject: Rebuttal Letter**

Please be informed that the authors acknowledge the time-taking and guiding suggestions of the reviewers and the editor. We believe that the valuable comments can make the paper more effective in terms of publication and readiness. We have meticulously reviewed and addressed each comment, and all modifications have been highlighted in the revised manuscript for ease of reference. We humbly request your reconsideration of our work and eagerly await your further feedback.The required documents are attached to the submission.

**1.0 Reviewer #1**

**Authors’ Reply:** Thanks for the valuable feedback. The robustness test has been added in heading 4.3. Moreover, the notes for the required tables have been added.

Reviewer #1: I did not see the robust tests in my 5th comment and even the authors did not reply to this comment. If robust tests are not added, readers will find it difficult to believe that the empirical results are reliable. The notes section of the tables have not been added, and without notes, how do readers know what ** *, * *, and * represent. Please make modifications according to the comments, and if you are unable to make the necessary changes, please inform us of the reason.


**2.0 Reviewer #2**

Reviewer #2: I have carefully reviewed your manuscript, taking into consideration the revisions made in response to the previous round of feedback. However, I have identified several issues that require further attention and improvement to ensure the quality of your research. Here are my review comments:

1.Your abstract includes the research background, research methods, and research findings but lacks a clear explanation of the research innovation or novelty. I recommend providing a clear statement of your research question and the unique contribution of your study in the abstract to capture the reader's interest.

**Authors’ Reply:** Abstract has been updated *‘This research fills the gap in existing literature by testing and implying the mediating role of Stakeholders’ Influence on the relationship between Green Banking Practices and the Bank’s Performance.’*

2.In your paper, there is insufficient explanation regarding the dimensions and items selected for your variables. Please provide more detailed information, explaining the theoretical basis and rationale behind your variable selection, to help readers better understand your research design.

**Authors’ Reply:** The items and variables are adapted form the following sources. *‘A structured questionnaire was used to obtain primary data from bankers from various banks [19,37,40,54]’*

3.You mention four types of stakeholders-competitors, consumers, shareholders, and top management of the organization, but you only collect data from top management. I suggest that you discuss in detail the roles of the other three types of stakeholders in your research and explain why you chose not to collect data from them or provide valid reasons for this decision.

**Authors’ Reply:** The authors take this comment as valuable in designing their future research. We believe that we can do further research and discuss the role of each stakeholder in detail. For this work the general role of investigated using the viewpoint of the banking personnel.

4.The absence of a table displaying model fit indices such as RMSEA, CFI, and TLI makes it impossible to assess the quality of model fit. Please include the results of these indices and explain their significance and relevance to your study.

**Authors’ Reply:** CFA table has been added under the heading 4.4 and all the values have been provided.

5.The factor loading threshold you use in the paper (greater than 0.4) is lower than the typically required threshold (greater than 0.7). Please provide a theoretical basis or prior research support for your choice of this lower threshold and ensure its validity and interpretability.

**Authors’ Reply:** The individual values of 0.4 have not been dropped as the collective sum of cronbach’s alpha using these item loading was more than 0.6.  *The items used to represent constructs pose satisfactory internal consistent relationships and a Cronbach’s alpha value of i.e. >0.5 [58]. The low factor loadings collectively gave a good value of Cronbach’s alpha therefore, items are not dropped. Reference provided.*

6.Your third research objective is to determine the role of stakeholders in the implementing of green banking guidelines, but the paper mainly focuses on interviews with banking personnel. Please specify how you plan to represent the roles of the other three types of stakeholders or provide a valid explanation for not studying them.

**Authors’ Reply:** The research aims to identify the role of stakeholders from the viewpoint of banking personnel . Their opinions are incorporated to check whether stakeholders are an important component in implementation of GB and have a role in improving Bank’s performance or not.

7.Have you considered conducting robustness checks to enhance the reliability of your research? If so, please provide details and results of any robustness checks you have conducted.

**Authors’ Reply:** The robustness test has been added in heading 4.3

In summary, your research has the potential to make a valuable contribution, but further revision and improvement are necessary to meet the standards of academic publishing. Please address and elaborate on the above issues in your revised manuscript to facilitate the continuation of the review process.

I wish you success in completing your revision work and look forward to seeing your final manuscript.

The authors thank the reviewers for their detailed suggestions and hope that this step will make the manuscript closer to publication and help in the contribution to the research and academia.

Regards,

Hammna Jillani

hammnajillani@gmail.com
