# Supplementary material for: The mediating role of stakeholders on green banking practices and bank’s performance: The case of a developing nation
Source: PLoS One. 2024 May 21;19(5):e0300585. doi: 10.1371/journal.pone.0300585 (PMC11108172; doi:10.1371/journal.pone.0300585)
Supplement: Attachment — Submitted filename: Response to reviewers.docx [file pone.0300585.s003.docx]

Dear Editor,

PLOS One.

**Subject: Rebuttal Letter**

Please be informed that the authors acknowledge the time-taking and guiding suggestions of the reviewers and the editor. We believe that the valuable comments can make the paper more effective in terms of publication and readiness. We have meticulously reviewed and addressed each comment, and all modifications have been highlighted in the revised manuscript for ease of reference. We humbly request your reconsideration of our work and eagerly await your further feedback.The required documents are attached to the submission.

Reviewer #1: The authors need to pay attention to the design of tables and the figure. For example, Figure 1, which is not very clear if directly used the original figure of the software.

**Authors reply: The size ratio has been increased.**

Reviewer #2: I have carefully reviewed the revised version of the manuscript. Although the manuscript is improved, it have not addressed the issues that I have mentioned before. And the revised version is confused to read, it is unclear which are revised compared with the previous version. In summary, I think this manuscript have not meet the standards of academic publishing, and recommend to reject it.

Reviewer #3: The idea of the paper is very good and in accordance with current market hot issues. However, here some issues are identified, If the author incorporate these points then this paper can be accepted.
1.There is a weak linkage between different parts of the paper. Particularly in the literature review, the author introduces four stakeholder groups, namely competitors, consumers, shareholders, and top organizational executives. While the subsequent analysis only involves the government and banks.

**Authors reply: The perspective of bankers is used to identify the role of stakeholders. The analysis focuses on the bankers; perspective only. An individual analysis of the stakeholders can be done as a separate study.**

2. The hypotheses are given without any references or theoretical backgrounds so there must be sound theoretical linkage with hypothesis.

**Authors reply: The references for hypotheses have been added.**

**2.4.1 Green Banking and Bank’s Performance**

Shaumya and Arulrajah [37] found a significant and positive relation between green banking practices on the environmental performance of the bank in Sri Lanka, thus indicating that the performance is increased if green practices are followed. In short, all the activities should be combined to foster the environmental growth of a bank. The bank’s employee-related, customer-related, operational, and policy-related activities have a direct influence on the implementation of green banking policy and green financing thus improving the environmental performance of the bank. Moreover, energy-efficient equipment and a well-designed environmental policy along with employee training and awareness sessions also contribute to the bank’s environmental performance. In Bangladesh, green funding is promoted to expand the environmental performance of the banks consequently leading to the economic development of the country [38, 39]. Choudhury et al., [40] suggest that greening the banking sector and taking an environmentally proactive approach can result in functional improvements and operational efficiencies in the banking sector. Furthermore, the bank’s image can be enhanced if green practices are adopted [41,42]. The literature backs that adopting green banking practices can lead to the financial, operational, and environmental performance of the bank [43]. Henceforth, the following hypothesis is proposed.

**H1:** There is a significant relationship between Green banking practices and the Bank’s performance.

**2.4.2 Stakeholders and Bank’s Performance**

In the 20th century, stakeholders started recognizing that environmental degradation and natural resource degeneration are the greatest externalities being produced by organizations, through operation and business activities. This resulted in increasing stakeholder pressure on organizations to reduce their adverse environmental impacts. Due to the formation of environmental conferences and international protocols, excessive pressure through external groups was formed to conserve the environment and incorporate sustainability in business operations [16]. In the beginning, the banking sector was not included in the organizations that harm the environment directly and require moderation in the policies and procedures but later on, the indirect impacts of the banking sector were identified and this sustainable finance and banking came into action.

For the adoption of green banking policy, pressure from all the stakeholder groups and international organizations has a direct influence. Moreover, social pressure plays a chief role in attaining sustainable growth and development of an organization. According to the stakeholder's theory, all the groups must be equally involved, not just the financers, to make the system grow successfully and work efficiently [44, 45]. The Stakeholder Theory projects that an organization occurs for the profit and benefit of numerous stakeholders moreover it produces externalities through its business activities that can affect stakeholders [46]. As a consequence of these externalities, there is the proliferation of stakeholder pressures on firms to shrink their negative impacts. Similar to the Stakeholder Theory, the Institutional Theory also supports the stakeholder approach by arguing that creating stakeholder engagement has become essential for organizations to establish social acceptability and competitiveness along with social sustainability [47].

There exist many groups of stakeholders including media, special interest groups, employees, research community government, etc. but in the literature, four major stakeholder groups are identified that have a direct impact and influence the speeding up of the development process in any organization. The four main groups are competitors, consumers, stockholders, and top management of the organization. All the shareholders play an important role in the development of green banking procedures and activities and stakeholders should be kept on the same page through effective communication by the management of any bank [40].

According to a study by Mehedi et al., [48], the organizational pressure of various stakeholders and environmental policy has the highest influence on any organization to develop and improve sustainability in business. Shafique and Majeed [41], explored the bankers’ intention to adopt green banking which can be influenced by Policy Guidelines, Attitude towards usage, Central Bank Regulations, and Management commitment and support. Linh and Anh [49], used a mixed methods approach of the questionnaire and in-depth interviews by the bank officials and the findings show the immense importance of stakeholders on green banking. Moreover, the study highlighted various benefits as green banking improves ties between local and international organizations, provides new opportunities for business growth thus minimizing capital losses, and engages all the stakeholders for organizational growth. This leads to the development of the following hypothesis.

**H2:** There is a significant relationship between Green banking practices and Stakeholders’ Influence.

**H3:** Stakeholders’ influence mediates the relationship between Green banking practices and the Bank’s performance.

3. The language of the introduction section is poor grammatically so it should be improved.

**Authors reply: The introduction has been revised.**

4.The sample size of the paper is close to the lower limit of the SEM model’s requirements. An insufficient sample can result in biases to the research findings. So, it should be increased.

**Authors reply: The sample size has been used as per reference paper.**

References: 19,37,40,54

5.Only a global Cronbach’s alpha index without displaying the latent variable-specific indices can affect the data’s credibility. The presence of items with low factor loadings in the factor loading table needs to be addressed by the author to bolster the persuasiveness of the study’s outcomes.

**Authors reply: The low factor loading values when combined give a good Cronbach’s alpha value, therefore retained.**

The items used to represent constructs pose satisfactory internal consistent relationships and a Cronbach’s alpha value of i.e. >0.5 [58]. The low factor loadings collectively gave a good value of Cronbach’s alpha therefore, items are not dropped.

6.The robustness test is absent, has the author considered incorporating robustness tests to enhance the study’s reliability? If not u should add in the study otherwise there will be question mark on the credibility of the results.

**Authors reply: The robustness test has been added.**

**4.3 Assessment of Non-Linearity**

Table 4 indicates the result of Ramsey’s RESET to assess the non-linearity. It is indicated that there exists no partial regression of BP on SI and GB. The results of bootstrapping indicate that neither of the nonlinear effects is significant thus indicating that the linear effects model is robust.

| **Non- Linear Relationship** | **Coefficient** | **p value** | **f** | **Ramsey’s RESET** |
| --- | --- | --- | --- | --- |
| GB*GB-> SI | 0.051 | 0.387 | 0.002 | F(3,242)= 0.97, p=0.598 |
| GB*GB -> BP | 0.035 | 0.490 | 0.001 |  |
| SI*SI -> BP | 0.023 | 0.448 | 0.001 |  |

Table 4: Non-Linearity Test

The authors thank the reviewers for their detailed suggestions and hope that this step will make the manuscript closer to publication and help in the contribution to the research and academia.

Regards,

Hammna Jillani

hammnajillani@gmail.com
